# Molecular and Pathological Heterogeneity of Synchronous Small and Large Duct Intrahepatic Cholangiocarcinoma—A Case Series

**DOI:** 10.3390/curroncol32050255

**Published:** 2025-04-27

**Authors:** Savelina Popovska, Vladislav Nankov, Boriana Ilcheva, George Dimitrov

**Affiliations:** 1 Department of Clinical Pathology, Medical University of Pleven, 5800 Pleven, Bulgaria; savelina.popovska@mu-pleven.bg; 2 Centre of Competence in Personalized Medicine, 3D and Telemedicine, Robotic Assisted and Minimally Invasive Surgery-Leonardo da Vinci, 5800 Pleven, Bulgaria; vladislav.nankov@mu-pleven.bg; 3 Department of Anatomy, Histology, Cytology and Biology, Medical University of Pleven, 5800 Pleven, Bulgaria; 4 Department of Pathology, Military Medical Academy, 1606 Sofia, Bulgaria; boryana_ilcheva@vma.bg; 5 Department of Medical Oncology, Medical University of Sofia, University Hospital “Tsaritsa Yoanna”, 1527 Sofia, Bulgaria; 6 Department of Nuclear Medicine, Radiotherapy and Medical Oncology, Medical University of Sofia, 1431 Sofia, Bulgaria

**Keywords:** intrahepatic cholangiocarcinoma, synchronous iCCA, small-duct, large-duct, molecular heterogeneity, targeted therapy, immune checkpoint inhibitors

## Abstract

Background: Synchronous small- and large-duct intrahepatic cholangiocarcinoma (iCCA) represents a rare and heterogeneous entity, posing challenges for diagnosis, prognosis, and treatment selection. The pathological and molecular diversity between these subtypes influences tumor behavior and therapeutic response, necessitating a personalized approach. This study investigates the molecular and pathological heterogeneity of synchronous iCCA and its clinical implications. Methods: This prospective case series included six patients diagnosed with synchronous small- and large-duct iCCA at the Military Medical Academy, Sofia, between January 2023 and January 2025, with a median follow-up of 15 months. Tumor classification was based on histopathological examination, immunohistochemical analysis, and next-generation sequencing (NGS)-based genomic profiling. Radiological and clinical data were analyzed to assess tumor growth patterns, treatment response, and progression-free survival (PFS). Results: Small-duct-predominant iCCA was associated with *IDH1/2* mutations and *FGFR2* fusions, a mass-forming growth pattern, and longer PFS. In contrast, large-duct-predominant iCCA exhibited *KRAS*, *TP53*, and *NF1* mutations, an infiltrative periductal growth pattern, and a more aggressive clinical course with shorter PFS. Tumor mutational burden-high (TMB-H) and microsatellite instability-high (MSI-H) were observed in a subset of large-duct iCCA cases, suggesting potential benefit from immune checkpoint inhibitors (ICIs). Conclusions: Synchronous small- and large-duct iCCA demonstrates distinct molecular, histopathological, and clinical features, necessitating individualized treatment strategies. Targeted therapies for *IDH1/2*- and *FGFR2*-altered small-duct iCCA have shown efficacy, whereas large-duct iCCA remains more aggressive and treatment-resistant, requiring novel therapeutic approaches. Future research should focus on adaptive treatment strategies that account for tumor heterogeneity and dominant molecular drivers.

## 1. Introduction

Intrahepatic cholangiocarcinoma (iCCA) is a rare primary liver malignancy that exhibits significant histological and molecular heterogeneity. It is traditionally classified into small-duct and large-duct subtypes, which differ in their molecular landscape, histopathology, clinical behavior, and therapeutic response [1]. However, an emerging subset of cases presents with synchronous small-duct and large-duct iCCA, where both subtypes coexist within the same tumor or patient, further complicating classification and treatment strategies [2].

Small-duct iCCA arises from the peripheral bile ducts and is characterized by a mass-forming growth pattern, non-mucin-producing cuboidal cells, and frequent association with chronic liver diseases, including hepatitis B or C and non-alcoholic fatty liver disease [3]. This subtype is commonly driven by *IDH1/2* mutations, *FGFR2* fusions, and *BAP1* alterations, which provide opportunities for targeted therapy [4]. In contrast, large-duct iCCA originates from larger bile ducts and exhibits an infiltrative, duct-forming growth pattern with mucin-producing columnar cells and a dense fibrotic stroma. It is often associated with primary sclerosing cholangitis and liver fluke infections and is molecularly characterized by *KRAS*, *SMAD4*, and *TP53* mutations [5].

Molecular profiling has revealed that these subtypes respond differently to therapy. Small-duct iCCA is frequently sensitive to IDH inhibitors and FGFR inhibitors [6,7], while large-duct iCCA, due to *KRAS*-driven oncogenesis and an immunosuppressive tumor microenvironment, remains more resistant to targeted therapies [8,9]. Furthermore, large-duct iCCA is generally more aggressive, with a higher incidence of lymph node metastasis, vascular invasion, and postoperative recurrence, leading to poorer overall survival compared to small-duct iCCA [10,11].

The presence of synchronous small-duct and large-duct iCCA within the same tumor or patient raises important questions about tumor evolution, molecular crosstalk, and treatment resistance mechanisms. Histopathological examination and immunohistochemical staining can confirm the coexistence of these subtypes, and recent studies suggest that they may exhibit distinct genetic alterations, reinforcing their biological differences [2]. However, molecular and clinical overlaps exist, complicating subclassification and therapeutic decision-making [12].

This case-series aims to analyze the molecular heterogeneity between synchronous small-duct and large-duct iCCA, correlating genomic alterations with histopathology and clinical outcomes.

## 2. Materials and Methods

### 2.1. Study Design and Patient Selection

This single-center prospective case series examines the molecular and pathological heterogeneity of synchronous small-duct and large-duct intrahepatic cholangiocarcinoma (iCCA). Among 87 patients diagnosed with iCCA, 6 cases with histopathologically confirmed synchronous small- and large-duct components were prospectively included at the Military Medical Academy, Sofia, between January 2023 and January 2025, with a median follow-up of 15 months.

For the purpose of this study, the term “synchronous small- and large-duct intrahepatic cholangiocarcinoma” was used to define cases in which both histologically distinct subtypes—small-duct and large-duct—were diagnosed concurrently, either within the same tumor in spatially distinct regions or in separate intrahepatic lesions. This classification was based on the identification of two morphologically and immunophenotypically distinct components, each displaying characteristic features consistent with established criteria for small-duct or large-duct iCCA. In contrast, “mixed small- and large-duct iCCA” typically refers to tumors with intermingled or overlapping histological features, where the two subtypes cannot be clearly separated. In this study, only cases with clearly distinguishable and co-existing subtypes were included under the definition of synchronous iCCA.

Diagnosis was established through histopathological evaluation and comprehensive genomic profiling, following the World Health Organization (WHO) tumor classification system, to assess the molecular and clinical significance of these distinct subtypes [13].

Patients were eligible if they had a histologically confirmed diagnosis of synchronous small-duct and large-duct iCCA, identified within the same tumor or at different intrahepatic sites. Tumor classification was based on morphological, immunohistochemical, and molecular criteria, ensuring accurate subclassification. Inclusion required sufficient tumor tissue for immunohistochemical staining and next-generation sequencing (NGS)-based genomic analysis. Patients were excluded if they exhibited only a single histological subtype or had undergone systemic therapy before resection, which could introduce confounding molecular alterations. Cases with insufficient tumor material for genomic sequencing were also excluded.

### 2.2. Histopathological and Immunohistochemical Analysis

Tumor specimens were obtained from surgical resections and were formalin-fixed and paraffin-embedded (FFPE) for histopathological and immunohistochemical analysis. Hematoxylin and eosin (H&E) staining was performed to classify tumors into small-duct and large-duct subtypes.

Small-duct iCCA was defined by non-mucin-producing cuboidal epithelial cells, duct-like structures, and a mass-forming growth pattern. In contrast, large-duct iCCA was identified by mucin-producing columnar cells, a duct-forming architecture, and a dense desmoplastic stroma. Cases with a mixed morphology were carefully evaluated to confirm the presence of both subtypes within the same tumor or different intrahepatic regions. The predominant component was defined as the histological subtype, occupying more than 50% of the tumor mass, as assessed on H&E-stained slides at x10 magnification. Radiological features were used to support the classification [14].

**Figure 1:**
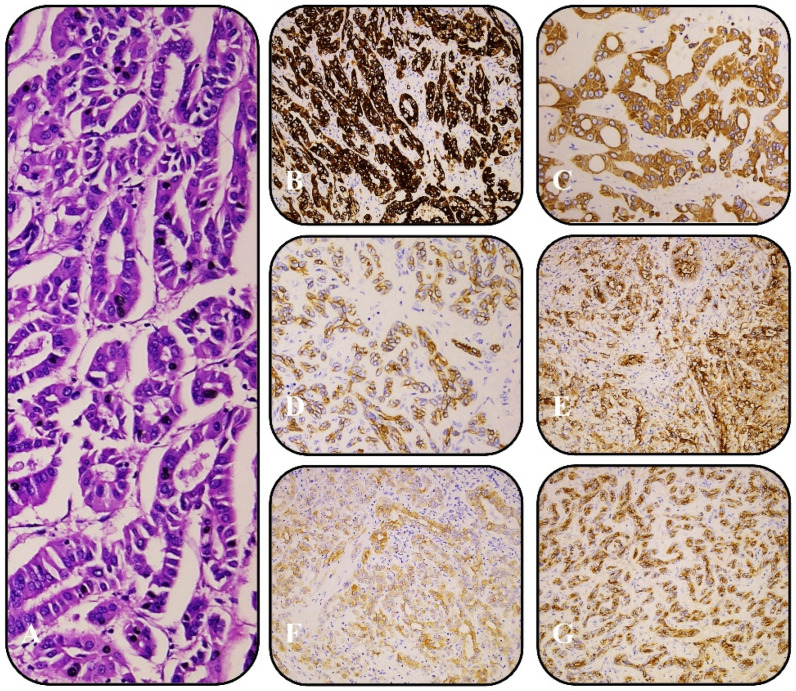
(**A**) H&E of large-duct iCCA; (**B**) positive IHC for CK7; (**C**) positive IHC for CK19; (**D**) positive IHC for EMA; (**E**) positive IHC for MOC-31; (**F**) positive IHC for S100P; (**G**) positive IHC for Villin. All samples are at ×10 magnification.

**Figure 2:**
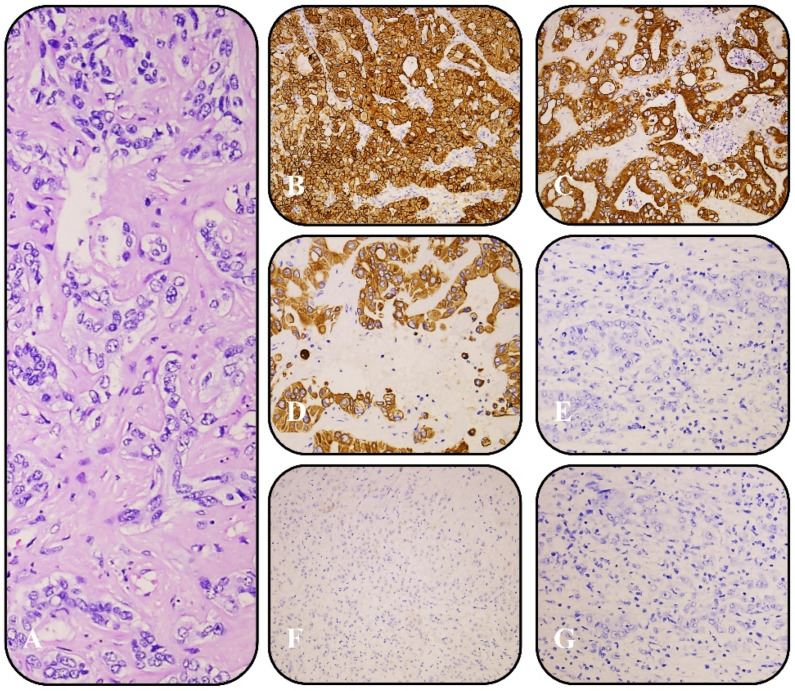
(**A**) H&E of small-duct iCCA; (**B**) positive IHC for CK7; (**C**) positive IHC for CK19; (**D**) positive IHC for EMA; (**E**) negative IHC for MOC-31; (**F**) negative IHC for S100P; (**G**) negative IHC for Villin. All samples are at ×10 magnification.

Immunohistochemistry (IHC) was performed on all cases to characterize tumor subtypes and distinguish iCCA from hepatocellular carcinoma. Small-duct iCCA typically exhibited strong positivity for CK7 (clone RNS, Leica/BOND), CK19 (clone D170, Leica/BOND), and EMA (clone GP1.4, Leica/BOND) (Figure 1), while large-duct iCCA was positive for CK7, CK19, EMA, MOC-31 (clone MOC31, Biocare/BOND), S100P, and Villin (clone 1d2C3, DAKO) (Figure 2). Additional markers, including HepPar-1 (clone OCH1E5, Leica/BOND), Arginase-1, and Glypican-3 (clone 1G12, Leica/BOND), were used to exclude hepatocellular carcinoma. Staining was performed on the Leica BOND and DAKO platforms using pre-diluted, ready-to-use antibodies according to the manufacturers’ protocols. The intensity and percentage of positive tumor cells were assessed by two independent pathologists to ensure diagnostic accuracy and reproducibility.

### 2.3. Molecular Profiling and Genomic Analysis

Comprehensive genomic profiling was conducted using TruSight™ Oncology 500 (Illumina, San Diego, CA, USA), a hybrid capture-based NGS panel covering 523 cancer-associated genes. DNA and RNA were co-extracted from FFPE tumor samples using the AllPrep^®^ DNA/RNA FFPE Kit (Qiagen, Hilden, Germany). The concentration and quality of the DNA isolates were assessed with the Qubit™ dsDNA HS Assay Kit (Invitrogen, Carlsbad, CA, USA) and the Infinium HD FFPE QC Assay Kit (Illumina, San Diego, CA, USA), respectively. RNA quantification was carried out with the Qubit™ RNA HS Assay Kit (Invitrogen, Carlsbad, CA, USA). Genomic DNA (gDNA) was sheared using a Covaris M220 Focused-ultrasonicator (Covaris, Woburn, MA, USA) in accordance with the manufacturer’s instructions. Libraries were sequenced on a NextSeq 550 instrument (Illumina, San Diego, CA, USA) to assess single nucleotide variants (SNVs), copy number variations (CNVs), structural rearrangements, gene fusions, and gene signatures [tumor mutational burden (TMB) and microsatellite instability (MSI)].

The genomic analysis focused on key alterations associated with cholangiocarcinoma, including *IDH1/2*, *FGFR2*, *KRAS*, *TP53*, *BAP1*, *NF1*, *MYCN*, *BRCA2*, and *ARID1A* mutations. TMB was quantified as the number of somatic mutations per megabase (mut/Mb), and MSI status was determined using an NGS-based MSI assay. Variants were classified according to clinical significance using OncoKB guidelines, where Tier I alterations were those with established clinical utility for targeted therapies, Tier II mutations were considered potentially actionable, and Tier III alterations were of uncertain significance [15].

### 2.4. Imaging Analysis and Clinical Correlation

All patients underwent contrast-enhanced computed tomography (CT) or magnetic resonance imaging (MRI) to evaluate tumor morphology, vascular invasion, and intrahepatic tumor distribution. Radiological features were analyzed to assess correlations between tumor growth patterns, molecular alterations, and histopathological classification.

Small-duct iCCA predominantly appeared as a well-defined, mass-forming lesion, whereas large-duct iCCA was more frequently associated with periductal infiltrative growth, biliary obstruction, and a dense fibrotic response. Imaging findings were compared with genomic alterations to identify potential associations between tumor morphology and molecular heterogeneity.

Clinical records were reviewed for tumor staging (TNM classification 8^th^ edition), treatment history, and response to therapy. Follow-up data, where available, were analyzed to assess disease progression and survival outcomes, providing insight into the prognostic differences between synchronous small- and large-duct iCCA. Patients received treatment according to ESMO guideline recommendations [16], based on their respective eligibility criteria.

### 2.5. Statistical Analysis

Descriptive statistics were used to summarize histopathological, molecular, radiological, and clinical data. Continuous variables, such as age and progression-free survival (PFS), were reported as medians with interquartile ranges (IQRs). Categorical variables, including histological subtype and molecular alterations, were presented as frequencies and percentages.

### 2.6. Ethical Considerations

This study was approved by the Institutional Ethics Board of the Military Medical Academy, Sofia, and conducted in accordance with the ethical principles outlined in the Declaration of Helsinki. Written informed consent was obtained from all patients before participation, and data were anonymized to protect patient confidentiality.

## 3. Results

### 3.1. Patient Characteristics

Between January 2023 and January 2025, a total of 87 patients were diagnosed with iCCA at the Military Medical Academy, Sofia. Of these, 38 patients (43.7%) were classified as small-duct type, 43 (49.4%) as large-duct type, and 6 patients (6.9%) were identified as having synchronous small- and large-duct features based on histomorphology and immunohistochemistry. The median age at diagnosis was 72 years (range: 64–78 years), with a female predominance (5 out of 6 patients, 83%). In all cases, both histologically distinct components—small-duct and large-duct—were identified either within the same tumor or at separate intrahepatic sites, confirming the diagnosis of synchronous iCCA.

### 3.2. Histopathological and Immunohistochemical Findings

All six cases demonstrated distinct small-duct and large-duct histopathological features within the same tumor or different liver regions. Immunohistochemically, the large-duct component expressed CK7, CK19, EMA, MOC-31, S100P, and Villin, while the small-duct component was positive for CK7, CK19, and EMA.

### 3.3. Genomic Alterations and Molecular Heterogeneity

NGS revealed distinct mutational landscapes in the synchronous small-duct and large-duct components within the same patient (Table 1). Small-duct iCCA was frequently associated with *IDH1/2* mutations (2/3 cases, 66%) and *FGFR2* fusions (1/3 cases, 33%), supporting its previously established molecular profile. Additionally, *BAP1* mutations (1/3 cases, 33%) and *MYCN* amplifications (1/3 cases, 33%) were observed in small-duct iCCA, potentially indicating alternative oncogenic pathways. Large-duct iCCA exhibited a higher frequency of *KRAS* mutations (2/3 cases, 66%), *TP53* mutations (1/3 cases, 33%), and *NF1* alterations (1/3 cases, 33%), consistent with a more aggressive molecular phenotype. Tumor mutational burden (TMB) varied significantly between subtypes, with large-duct iCCA generally exhibiting a higher TMB (range: 3.1–23.5 muts/Mb) compared to small-duct iCCA (range: 1.6–7.8 muts/Mb). One case of large-duct iCCA exhibited high microsatellite instability (MSI-H, 14.8%), suggesting potential sensitivity to immune checkpoint inhibitors, whereas small-duct iCCA cases were predominantly MSI-low (range: 2.3–3.2%).

### 3.4. Imaging and Clinical Correlation

Initial radiological evaluation revealed distinct imaging patterns between small-duct and large-duct components. In five of the six cases, imaging findings correlated well with histological classification: small-duct predominant cases presented with mass-forming lesions, while large-duct predominant tumors exhibited infiltrative or periductal growth patterns. However, one case showed discordant features—although the radiological appearance suggested a small-duct pattern, histological analysis revealed a predominance of large-duct morphology. Tumor staging varied across the cohort, with two patients classified as Stage II and four as Stage IIIB. Among the six patients included in our study, all underwent curative-intent surgical resection. Three received adjuvant chemotherapy with capecitabine due to high-risk features (i.e., nodal involvement, T4 stage), while the remaining three were managed with active surveillance.

Small-duct iCCA lesions were primarily well-defined, mass-forming tumors with minimal biliary involvement, whereas large-duct iCCA displayed periductal infiltrative growth, biliary obstruction, and vascular invasion. Biliary obstruction and periductal infiltration were exclusive to large-duct components, correlating with *KRAS* and *TP53* mutations in this cohort. Small-duct iCCA tumors with *FGFR2* fusions were more likely to present as well-circumscribed, mass-forming lesions without significant ductal involvement. In contrast, patients with MSI-H large-duct iCCA exhibited highly infiltrative tumor morphology, with extensive lymph node involvement. Clinical follow-up revealed worse prognostic outcomes in cases with predominant large-duct components, particularly those harboring *KRAS*, *TP53*, and *NF1* mutations (Table 2).

## 4. Discussion

The management of synchronous small- and large-duct iCCA remains challenging due to its histopathological and high molecular heterogeneity. Treatment selection is largely determined by the predominant histological component, disease stage, and molecular profile. In this case series, patients with small-duct-predominant iCCA, particularly those harboring *IDH1/2* mutations or *FGFR2* fusions, exhibited longer progression-free survival (PFS), supporting the use of targeted therapies such as ivosidenib and pemigatinib, both of which are approved for advanced cholangiocarcinoma [16,17]. In contrast, large-duct-predominant iCCA, frequently associated with *KRAS*, *TP53*, and *NF1* mutations, demonstrated more aggressive behavior, higher recurrence rates, and shorter PFS. The absence of approved KRAS-targeted therapies in cholangiocarcinoma limits treatment options to gemcitabine/cisplatin chemotherapy, though emerging strategies, including MEK and SHP2 inhibitors, are under investigation [18,19]. Notably, *NF1*-mutant large-duct iCCA exhibited particularly poor outcomes, suggesting that RAS/MAPK or PI3K inhibitors could be explored in future clinical trials.

Immunotherapy, in the context of precision oncology, has emerged as a promising option for a subset of cholangiocarcinoma patients, particularly those with high tumor mutational burden (TMB-H) or microsatellite instability-high (MSI-H) status. TMB, defined as the total number of mutations per megabase of DNA, serves as an agnostic predictive biomarker for immune checkpoint inhibitor (ICI) response. TMB-H (≥10 mutations/Mb) has been associated with improved outcomes in patients receiving pembrolizumab [20]. Similarly, MSI-H status, which results from deficient mismatch repair (dMMR) mechanisms, enhances tumor immunogenicity and response to ICIs [21]. In the context of iCCA, tumors with TMB-H and/or MSI-H status have demonstrated improved outcomes from ICI-based therapies [22]. Despite this evidence, ICIs remain inaccessible in Bulgaria for TMB-H/MSI-H cholangiocarcinoma due to a lack of reimbursement policies, limiting their real-world application.

In addition to targeted therapy and immunotherapy, systemic chemotherapy remains the cornerstone of treatment for advanced cholangiocarcinoma. First-line therapy consists of gemcitabine and cisplatin, often combined with durvalumab, as demonstrated in the TOPAZ-1 trial [23]. The trial showed that the addition of durvalumab to chemotherapy modestly improved overall survival (OS) and PFS, with a PFS benefit of 1.5 months and an OS improvement of 1.3 months over the control arm. However, subgroup analysis revealed significant benefit primarily in PD-L1-positive and Asian patients, who comprised the majority of the study cohort, raising concerns about the generalizability of these findings and the need for more stringent approval criteria based on biomarker selection. For second-line therapy, FOLFOX (5FU/folinic acid-oxaliplatin) has shown modest survival benefits, but prognosis remains poor, with median survival for advanced disease under one year [16,17].

A key limitation of our study is the relatively short median follow-up period of 15 months, which hinders the ability to make definitive conclusions regarding long-term outcomes. However, this limitation is inherent in the design of our prospective case series, which focuses on a rare iCCA variant within a single specialized center. Given the exceptional rarity of synchronous small- and large-duct iCCA, the current follow-up duration reflects the early phase of patient accrual. For context, the TOPAZ-1 trial, which established current systemic treatment standards, reported a median follow-up of only 23 months [23].

Molecular heterogeneity within individual tumors complicates treatment selection, highlighting the importance of multi-regional tumor sampling and comprehensive genomic profiling [24]. A one-size-fits-all approach is insufficient, as treating iCCA without accounting for the dominant molecular driver may lead to suboptimal therapeutic responses and unnecessary financial burden [25]. Identifying subtype-specific oncogenic drivers is crucial for selecting the most effective targeted therapies and minimizing unnecessary interventions.

Histopathological complexity remains a significant challenge in the diagnosis and treatment of synchronous iCCA. The coexistence of small- and large-duct components within the same tumor complicates classification, requiring detailed histological and immunohistochemical analysis [14]. Radiological growth patterns are often used as non-invasive surrogates for tumor subtype classification in iCCA [26]; however, our findings emphasize the limitations of relying solely on imaging. In five of the six cases, radiological features were concordant with the predominant histological subtype, with mass-forming lesions observed in small-duct iCCA and periductal-infiltrative patterns typical of large-duct disease. Nonetheless, one case demonstrated a discordance between radiological and histological findings, underscoring the potential for misclassification based on imaging alone. This reinforces the critical role of histopathological and immunohistochemical evaluation in accurately identifying the predominant tumor component, particularly in heterogeneous or borderline cases, and in guiding appropriate therapeutic decision-making.

Surgical management also differs between subtypes. Large-duct iCCA frequently requires extensive resection, including lymphadenectomy and biliary reconstruction, due to its higher propensity for lymph node metastasis and vascular invasion. In contrast, small-duct iCCA is often more localized and may be resected with limited hepatectomy [27]. Prognostically, small-duct iCCA is associated with better survival outcomes, whereas large-duct iCCA exhibits early recurrence and poor overall survival, reinforcing the need for subtype-specific treatment strategies [28].

## 5. Conclusions

This case series underscores the importance of a personalized, multidisciplinary approach to synchronous iCCA, integrating histopathological classification, molecular profiling, and precision therapy to optimize patient outcomes. Expanding access to ICIs for MSI-H and TMB-H cases could significantly improve treatment outcomes for selected patients. Future research should focus on adaptive treatment strategies tailored to tumor heterogeneity and dominant molecular drivers, ensuring that patients receive the most effective therapy based on their tumor’s unique characteristics while simultaneously reducing unnecessary financial burdens.

## Figures and Tables

**Table 1:** Pathological characteristics.

Case	Sex	Age (Years)	Predominant Variant (%)	IHC	Small-Duct iCCA Mutations	Large-Duct iCCA Mutations	TMB (mut/Mb)	MSI Status
1	Female	72	Small-duct (65%)	—	*MYCN* amp, *BRCA2* fusion, *PIK3R1*	*KRAS*, *TP53*	7.8 (small)/3.1 (large)	MSI-L (3.2%)
2	Female	78	Small-duct (60%)	EMA+, CK7+, CK19+, Hepatocyte−, Arginase-1−	*IDH2*, *KRAS*, *EGFR* amp	*KRAS*	2.3 (small)/3.1 (large)	MSI-L (2.4%)
3	Female	77	Small-duct (85%)	CK7+, TTF1−, GATA3−, Hepatocyte−	*IDH1*, *EGFR* amp, *MDM2* amp	*KRAS*	1.6 (small)/3.1 (large)	MSI-L (2.9%)
4	Female	72	Large-duct (75%)	CK7+, CK20 (focal)+, ER−, TTF1−, GATA3−, CDX2−	*BAP1* mutation	*TP53*	7.9 (small)/23.5 (large)	MSI-L (1.2%)
5	Male	64	Large-duct (90%)	—	*ARID1A*, *ATM*	*NF1*	3.1 (small)/23.5 (large)	MSI-H (14.8%)
6	Female	65	Large-duct (55%)	CK7+, CK19+, CK8/18+, EMA+, Villin+, MOC-31+, Hepatocyte−, Glypican 3−, Arginase−, HMWCK−	*FGFR2* fusion	*KRAS*, *SMAD4*	2.9 (small)/7.8 (large)	MSI-L (2.9%)

Abbreviations: *MYCN*; avian myelocytomatosis viral oncogene neuroblastoma derived homolog, *BRCA2*; breast cancer gene-2, *PIK3R1*; phosphoinositide-3-kinase regulatory subunit-1, *IDH1/2*; isocitrate dehydrogenase-1/2, *KRAS*; Kristen Rat Sarcoma Viral oncogene homolog, *EGFR*; epidermal growth factor receptor-1, *MDM2*; murine double minute 2, *BAP1*; BRCA1 associated protein-1, *ARID1A*; AT-rich interaction domain 1A, *ATM*; ataxia telangiectasia mutated, *FGFR2*; fibroblast growth factor receptor-2, *TP53*; tumor protein-53, *NF1*; Neurofibromin-1, *SMAD4*; SMAD family member-4, TMB; Tumor mutational burden, MSI-L/H; microsatellite instability-low/high.

**Table 2:** Initial radiological findings and clinical outcomes.

Case	Localization	Biliary Obstruction	Vascular Invasion	Lymph Node Involvement	Grade and Stage	Adjuvant Treatment	PFS (Months)
1	Poorly defined right hepatic lobe lesion	No	No	No	G2; pT4N0M0/IIIB	Capecitabine	12, 10
2	Left hepatic lobe lesion (5.24 × 3.7 cm)	No	No	No	G3; pT2aN0M0/II	-	14, 50
3	Multiple lesions in segments IVb, V and VII	No	No	No	G2; pT2bN0M0/II	-	13, 80
4	Mass in segments VII–VIII (9.74 × 8.3 cm)	Yes	Yes	Yes	G2; pT4N1M0/IIIB	Capecitabine	5, 40
5	Hypervascular lesions in segments II-V and VIII	Yes	Yes	Yes	G3; pT2N1M0/IIIB	-	3, 70
6	Poorly defined lesion in segment IV with left hepatic duct invasion	Yes	Yes	Yes	G2; pT2N1M0/IIIB	Capecitabine	6, 20

Abbreviation: PFS; progression-free survival.

## Data Availability

The dataset presented in this article is not readily available due to patient privacy protection.
